# Special Issue “Recent Advances in Oral Drug Delivery Development”

**DOI:** 10.3390/ph16091289

**Published:** 2023-09-13

**Authors:** Joël Schlatter

**Affiliations:** Assistance Publique-Hôpitaux de Paris (AP-HP), Hôpital Paul Doumer, Service de Pharmacie, 60140 Labruyère, France; joel.schlatter@aphp.fr

This Special Issue, entitled “Recent Advances in Oral Drug Delivery Development”, aims to demonstrate new advances and future trends in the field of oral drug delivery. Although oral administration is the most commonly used treatment due to its obvious benefits, oral drug delivery may be limited by factors such as gastroduodenal anatomy, chemical substances interfering with bioavailability, and physiological characteristics unique to the individual [1,2,3]. Because the therapeutic efficacy of orally administered drugs is dependent on their physicochemical properties and biological interactions, novel approaches to drug delivery development, including nanocarriers, micelles, cyclodextrins, and lipid-based carriers, are currently being investigated to improve bioavailability and therapeutic activity [4].

Our call for papers for this Special Issue drew great interest from a broad spectrum of researchers working primarily in pharmaceutical technology, hospital pharmacy, clinical research, and basic medical research. A total of 40 authors from all over the world (Russia, South Africa, France, Pakistan, and Saudi Arabia) contributed to four research papers and one review. The published articles highlight various experimental or clinical aspects of oral drug delivery development to ensure appropriate medications.

Since marketed liquid formulations are not always available for younger pediatric patients, Annereau et al. described the development of an oral compounded suspension of temozolomide (TMZ) used as the standard of care for many pediatric brain tumors, such as neuroblastoma, glioblastoma, and medulloblastoma [5]. The authors underlined the role of pharmacists in compounding this liquid formulation because the available TMZ capsules are open, and the powder is mixed in a compote or drink, exposing caregivers and parents to this hazardous drug. The preparation was developed with the best compromise between chemical stability, dosing accuracy, volume minimization, palatability, and acceptability. Annereau et al., recognizing the real complexity of producing a stable liquid form, demonstrated TMZ stability in liquid formulation for 8 weeks under refrigerated conditions. However, the best challenge was to identify the best taste-masking agent using a new composite scale. The new available compounded TMZ formulation was evaluated in 104 patients over a 5-year period in a single cancer center.

Likewise, Secretan et al. proposed the development of a simple oral solution of Epigallocatechin gallate (EGCG) used to reduce oxidative damage to lipids, proteins, and DNA or to prevent tumor progression [6]. Here, the challenge was to stabilize EGCG in aqueous solution by using suitable excipients and controlling some process parameters to target these main factors responsible for catechin degradation. To enhance the stability, the complexation of the drug was used by investigating the physical change of the drug solutions spiked with carbohydrates such as sucrose. Experimental and theoretical studies have demonstrated the formation of an EGCG–sucrose complex that increases the drug’s stability in a liquid formulation. In this article, the characterization of the molecular interactions specific to an active ingredient could be considered to increase the solubility, stability, and bioavailability of the ingredient.

The third article is focused on the development of a mucoadhesive buccal delivery system for the sustained delivery of metformin (MET) and sitagliptin (SIT) against diabetes mellitus [7]. Currently, conventional dosage forms lead to uncontrolled drug release, instability, and less bioavailability of metformin. To ensure systemic drug release, Shakir et al. proposed the formulation of buccal mucoadhesive tablets with the combination of both drugs. The new formulation was investigated in vitro for mucoadhesion, strength, biocompatibility, hemocompatibility, and release profile, and in vivo for bioavailability. Buccal delivery provides some advantages. The oral cavity is more vascularized and accessible for administration, avoiding acid hydrolysis in the gastrointestinal tract and bypassing the “first-pass” effect [8].

Similarly, Seo et al. presented a review discussing the findings from studies focusing on oral lipophilic drug delivery systems, including self-emulsifying drug delivery systems (SEDDSs) for the treatment of uncomplicated malaria [9]. The authors detailed the concept of a SEDDS, which is used to solubilize drugs. Additionally, the advantages of SDEES and their limitations were included in the review. To overcome the limitations described, the authors proposed the current approach in research, including solidification techniques. Some important solidification techniques were highlighted, such as capsule filling, spray drying, spray cooling, melt granulation, melt extrusion, and freeze-drying. In conclusion, solid SEDDS appeared to have a bright future for enabling the incorporation of highly hydrophobic drugs into lipophilic drug delivery systems.

The last article focused on the evaluation of the subchronic toxicity of oral anthrafuran drug formulations in Chinchilla rabbits. This route of administration is becoming more popular among oncologists since it improves the quality of life of patients and reduces their time in the clinic [10]. The study showed that anthrafuran induced a reduction in body weight, which could be interpreted as indirect evidence of gastrointestinal toxicity, possible manifestations of hematologic and cardiologic toxic properties at high doses of drug, hepatotoxicity manifesting in an increase in transaminase levels, and nephrotoxicity depending on drug dose. Unlike doxorubicin, no testicular toxicity was observed after anthrafuran administration. The authors concluded that the toxicity of oral anthrafuran depended on dose, and multiple administrations of therapeutic doses of the drug produced transient, completely reversible toxic effects.

In summary, this Special Issue highlights and presents recent findings on oral drug delivery development. Considering these diverse contributions, the value of multidisciplinary collaboration, combining the expertise of laboratory researchers and clinicians, is obvious. Finally, the Guest Editor expresses heartfelt thanks to all authors for their valuable contributions and would also like to thank Ms. Evelyn Du for her assistance and technical support.

## Data Availability

Data sharing not applicable.
